# Complete mitochondrial and phylogenetic relationships of *Dermacentor everestianus* (Acari: Ixodidae) and *Alveonasus* sp. (Acari: Argasidae)

**DOI:** 10.1186/s12864-026-12933-2

**Published:** 2026-05-21

**Authors:** Xialing Zhao, Bin Shi, Wenqiang Tang, Zengqiang Liu, Chenyang Xia

**Affiliations:** Academy of Agriculture and Animal Husbandry Sciences, Key Laboratory of Animal Parasitosis of Xizang Autonomous Region, Xizang Academy of Agricultural and Animal Husbandry Sciences Institute of Animal Science, Lhasa, 850009 China

**Keywords:** Mitochondrial genome, Phylogenetic analysis, *Dermacentor everestianus*, *Alveonasus* sp.

## Abstract

**Background:**

Ticks are hematophagous arthropods capable of transmitting pathogens threatening human and animal health. The use of individual gene markers has limited phylogenetic studies of ticks, but the mitochondrial genome offers critical data for understanding phylogeny and molecular evolution.

**Results:**

The mitogenomes of *Dermacentor everestianus* and *Alveonasus* sp. were assembled from Illumina 350 bp pair-end reads. In this study, we sequenced the complete mitochondrial genomes of *Dermacentor everestianus* and *Alveonasus* sp., which measured 16,413 bp and 14,545 bp, respectively. Both mitogenomes contained 2 ribosomal RNA (rRNA) genes, 22 transfer RNA (tRNA) genes, and 13 protein-coding genes (PCGs). *Dermacentor everestianus* has two similar insertions between tRNA-Phe and tRNA-Gln and between tRNA-Glu and ND1. The adenine + thymine (A + T) content was 78.68% for *D. everestianus* and 71.60% for *Alveonasus* sp. *Dermacentor everestianus* showed negative AT- and GC-skews, whereas *Alveonasus* sp. exhibited positive AT-skews and negative GC-skews. The nucleotide diversity (Pi) and nonsynonymous (Ka)/synonymous (Ks) mutation rate ratios indicated that *NAD1*, *COX1*, and *COX3* were the most conserved genes, with *COX1* showing the lowest evolutionary rate. Phylogenetic analysis was performed using maximum likelihood (ML) methods based on *COI* gene and 13 PCGs. The results revealed that *D. everestianus* belongs to the Ixodidae family, within the *Dermacentor* genus, while *Alveonasus* sp. should be classified as a separate evolutionary lineage within the Argasidae family.

**Conclusions:**

These findings provide valuable insights into tick genetic variation, molecular classification, and evolutionary relationships, offering a foundation for future research in these areas.

**Supplementary Information:**

The online version contains supplementary material available at 10.1186/s12864-026-12933-2.

## Introduction

Ticks are ectoparasites that feed on vertebrates and are obligate blood-sucking arthropods. They serve as both carriers and reservoirs of significant pathogens that cause diseases in humans and animals, resulting in substantial economic losses in the livestock industry [1, 2]. Approximately 10% of ticks worldwide are reported to harbor pathogens, and they are recognized as the vectors with the highest number of disease transmissions [3]. Ticks are known to transmit up to 210 pathogens, including those responsible for *hemorrhagic fever* [4], *rickettsioses* [5], *Babesia ovata* [6], and *hantavirus diseases* [7], which has garnered increased attention in public health research [8]. Despite their critical role in disease transmission, the mitochondrial genomes of ticks have not been fully characterized, and their phylogenetic relationships remain unclear, particularly in regions like Xizang, China. *Dermacentor everestianus* and *Alveonasus* sp. are distributed in the Qinghai-Tibet Plateau and Xinjiang of China and Nepal [9]. Adult ticks typically infest large mammals such as yaks, horses, sheep, and goats, while larvae and nymphs feed on rodents [10]. The Qinghai-Tibetan Plateau (QTP), colloquially called ‘the roof of the world’, is the highest and largest plateau globally. With an average elevation of 4,500 m above sea level and covering an area of more than 2.5 million square kilometers, it exerts a profound influence on regional and global climate patterns [11]. Its complex inland ecological environment provides favorable conditions for tick survival. Previous studies have reported infection rates as high as 80% in Tibetan cattle and sheep, suggesting a wide spread prevalence of tick-borne infections. Thus, studying ticks in this region is of urgent importance. Mitochondria are essential organelles in eukaryotic cells, possessing their genome independent of the nuclear genome, known as the mitochondrial genome [12]. As a molecular genetic marker, the mitochondrial genome has been widely applied in insect phylogenetic studies due to its maternal inheritance, rapid evolutionary rate, and relatively conserved genomic structure [13, 14]. Although a mitochondrial genome of *D. everestianus* has been previously reported [15], the mitogenomic characteristics of this species as well as *Alveonasus* sp. in the Xizang region of China remain insufficiently explored.

In this study, next-generation sequencing was employed to determine the complete mitochondrial genome sequence and structure of *D. everestianus* and *Alveonasus* sp. We performed an in-depth examination of genome size, nucleotide composition, codon usage patterns, gene overlaps, intergenic regions, tRNA secondary structures, and the A + T-rich region. Additionally, the phylogenetic positions of *D. everestianus* and *Alveonasus* sp. were reassessed using Maximum Likelihood (ML) methods. These findings will contribute to a deeper understanding of mitogenome evolution and the phylogenetic relationships within the genus, particularly for *D. everestianus* and *Alveonasus* sp.

## Materials and methods

### Identification of ticks

Specimens of ticks were collected from Tibetan sheep in the Shigatse (29° 21′ 7″ N, 85° 3′ 36″ E) and Ali areas (32° 33′ 34″ N, 79° 55′ 37″ E) in Xizang, China, in 2023. Three to five ticks were collected from different parts of each sheep, and 30 ticks were randomly selected from each area for this study. Ticks were directly removed from the animals’ body surfaces using steel forceps. Notably, this non-invasive collection operation caused no physical damage or stress to the host sheep, and no experimental animals were anesthetized or rendered unconscious during the entire sampling process. The ticks were immersed in 75% ethanol and stored at 4 °C until analysis. Adult ticks were selected from the sample tube, and tick morphology was examined using a stereoscopic microscope. Tick species were identified using a tick identification key [16, 17], and various characteristics, including the dorsum, venter, basis capituli dorsum, basis capituli venter, scutum, and tarsus, were recorded.

### Mitogenome sequencing and assembly

Total genomic DNA was isolated utilizing the Genomic DNA Extraction Kit following the manufacturer’s instructions (Beijing Cowin Biotech Co., Ltd.). The integrity and purity of the extracted DNA were checked using 1% agarose gels, and DNA concentration was determined with the Qubit^®^ DNA Assay Kit in the Qubit^®^ 3.0 Fluorometer (Invitrogen, USA). A total of 0.2 µg of DNA per sample was utilized to prepare the sequencing library. The NEBNext^®^ Ultra™ DNA Library Prep Kit for Illumina (NEB, USA) was employed, and unique index codes were assigned to each sample. Genomic DNA was fragmented to an average size of 350 bp through sonication. The resulting fragments were end-polished, A-tailed, and ligated with full-length Illumina adapters before undergoing PCR amplification [18]. After PCR amplification, the products were purified by the Beckman Coulter AMPure XP system (Beckman Coulter, Beverly, USA), and the DNA concentration was measured by the Qubit^®^ 3.0 Fluorometer (Invitrogen, USA). The libraries were then analyzed for size distribution using NGS3K/Caliper and quantified by real-time PCR (3 nM). Genomic DNA library preparation and sequencing were carried out by Shenzhen Huitong Biotechnology (Shenzhen, China).

After filtering the raw data (10.72 Gb for *D. everestianus* and 10.38 Gb for *Alveonasus* sp.) using the NGS QC Toolkit, 10.15 GB and 9.44 Gb of clean data were obtained for *D. everestianus* and *Alveonasus* sp., respectively [19]. The Q20 and Q30 values were 96.6% and 92.49% for *D. everestianus*, and 95.34% and 90.72% for *Alveonasus* sp., indicating high sequencing quality. The mitochondrial genomes were assembled into single, circular contigs with high coverage, and their conserved gene structure further supported the reliability of the assembly. The completeness of the mitogenomes was validated and refined by visualizing the GFA graph files generated by SPAdes v3.11.0 [20].

### Genome annotation and sequence analysis

The mitochondrial genomes of *D. everestianus* and *Alveonasus* sp. were annotated for protein-coding genes and tRNA utilizing the MITOS software (http://mitos.bioinf.uni-leipzig.de/index.py). The rRNA genes were predicted following established methods [21, 22]. A circular mitogenome map was generated with OrganellarGenomeDRAW v1.2 [23]. Base composition, codon usage, relative synonymous codon usage (RSCU), and amino acid content were calculated utilizing MEGA v.7.0.26 [24]. UNucleotide bias was measured with the formulas AT-skew = [A − T]/[A + T] and GC-skew = [G − C]/[G + C] [25]. The nucleotide diversity (Pi) and nonsynonymous (Ka)/synonymous (Ks) mutation rate ratios of 13 PCGs were analyzed with DnaSP v5.10.01 [26].

### Cytochrome oxidase I phylogenetic analysis

The *COI* genes of ticks species were obtained from the GenBank database to construct a phylogenetic tree. *Nuttalliella namaqua* was used as the outgroup. Phylogenetic analyses were then conducted using ML inference with IQ-TREE 1.6.10 [27]. The confidence levels of the branching nodes of IQ-TREE were estimated using the ultrafast bootstrap approximation approach (UFBoot) (10,000 repetitions). Sequence similarity and alignment analysis of the *COI* gene from *Alveonasus* sp. on the monophyletic branch was conducted using MegAlign within the DNASTAR software package.

### Mitochondrial genome phylogenetic analysis

The complete mitochondrial genome sequences of ticks species were obtained from the GenBank database to construct a phylogenetic tree (Table 1). *Nuttalliella namaqua* was used as the outgroup. Based on the 13 protein-coding genes (PCGs), Phylosuite software, in combination with MACSE v2 [28], was used to perform multiple alignments and concatenation to generate the nucleotide sequence data for these genes. Phylogenetic analyses were then conducted using ML inference with IQ-TREE 1.6.10. The confidence levels of the branching nodes of IQ-TREE were estimated using the ultrafast bootstrap approximation approach (UFBoot) (10,000 repetitions). Sequence similarity and alignment analysis of the 13 PCGs from *Alveonasus* sp. on the monophyletic branch was conducted using MegAlign within the DNASTAR software package.

**Table 1:** Detailed sequence information of mitochondrial genomes used in this study

Family	Genus	Species	Genbank accession	Length(bp)
*Argasidae*	*Ornithodoros*	*Ornithodoros sonrai*	NC039688	14,430
		*Ornithodoros brasiliensis*	NC023373	14,489
		*Ornithodoros waterbergensis*	KR907251	14,400
		*Ornithodoros moubata*	NC004357	14,398
		*Ornithodoros hermsi*	NC039832	14,430
		*Ornithodoros porcinus*	NC005820	14,378
		*Ornithodoros zumpti*	KR907257	14,438
		*Ornithodoros compactus*	KY457534	14,400
		*Ornithodoros costalis*	KJ133591	14,442
		*Ornithodoros rostratus*	NC023372	14,452
	*Alectorobius*	*Alectorobius capensis*	NC005291	14,418
		*Alectorobius amblus*	ON756083	14,401
		*Alectorobius spheniscus*	ON756087	14,482
		*Alectorobius microlophi*	ON800863	14,409
		*Alectorobius atacamensis*	ON800845	14,430
		*Alectorobius atacamensis*	ON800845	14,430
		*Alectorobius rioplatensis*	ON800876	14,451
		*Alectorobius guaporensis*	ON800857	14,416
		*Alectorobius cerradoensis*	ON800849	14,415
		*Alectorobius puertoricensis*	ON800872	14,424
	*Argas*	*Argas africolumbae*	NC019642	14,440
		*Argas lagenoplastis*	NC023369	14,478
		*Argas* sp.	KC769588	14,450
		*Argas ricei*	NC067900	14,450
		*Argas miniatus*	NC023371	14,416
		*Argas persicus*	NC053794	14,426
		*Argas walkerae*	NC037523	14,437
	*Navis*	*Navis striatus*	NC037522	14,487
	*Ogadenus*	*Ogadenus brumpti* ssp.	KY457510	14,519
	*Secretargas*	*Secretargas transgariepinus*	KY457523	14,534
	*Otobius*	*Otobius lagophilus*	ON800860	14,535
	*Alveonasus*	*Alveonasus lahorensis*	PP072240	14,507
		*Alveonasus lahorensis*	PP072241	14,507
	*Chiropterargas*	*Chiropterargas confusus*	KY457514	14,287
		*Chiropterargas boueti*	NC056777	14,307
	*Carios*	*Carios vespertilionis*	NC060373	14,521
	*Nothoaspis*	*Nothoaspis amazoniensis*	NC033900	14,416
	*Reticulinasus*	*Reticulinasus faini*	NC037524	14,433
*Ixodidae*	*Dermacentor*	*Dermacentor auratus*	MW034677	14,766
		*Dermacentor everestianus*	NC042764	15,191
		*Dermacentor marginatus*	MK905212	15,067
		*Dermacentor nitens*	NC023349	14,839
		*Dermacentor nuttalli*	NC028528	15,086
		*Dermacentor reticulatus*	MT478096	14,806
		*Dermacentor rhinocerinus*	KY457527	14,708
		*Dermacentor silvarum*	NC026552	14,945
	*Hyalomma*	*Hyalomma truncatum*	KY457529	14,731
		*Hyalomma anatolicum*	MW546283	14,731
		*Hyalomma excavatum*	MW546284	14,733
		*Hyalomma marginatum*	MN885800	14,764
	*Haemaphysalis*	*Haemaphysalis sulcata*	NC062063	14,679
		*Haemaphysalis doenitzi*	NC062158	14,671
		*Haemaphysalis hystricis*	NC039765	14,716
		*Haemaphysalis longicornis*	NC037493	14,718
	*Rhipicephalus*	*Rhipicephalus decoloratus*	NC052828	15,268
		*Rhipicephalus evertsi*	KY457537	14,739
		*Rhipicephalus simus*	KJ739594	14,929
		*Rhipicephalus zambeziensis*	KY457543	14,691
		*Rhipicephalus turanicus*	NC035946	14,717
	*Ixodes*	*Ixodes hexagonus*	AF081828	14,539
		*Ixodes pavlovskyi*	KJ000060	14,575
		*Ixodes persulcatus*	NC004370	14,539
		*Ixodes ricinus*	NC018369	14,566
		*Ixodes holocyclus*	MH043267	15,007
		*Ixodes uriae*	NC006078	15,053
		*Ixodes tasmani*	NC041086	15,227
	*Nuttalliella*	*Nuttalliella namaqua*	JQ665719	14,425

## Results

### Morphological features of ticks

The body of *D. everestianus* was ovoid, with a short, rectangular capitulum. The palps were stubby, with palp articles II and III equal in width. The tarsus I had a short dorsal distance, and the posterior margin of segment II was not visible. Each leg coxa had enamel spots, with 11 festoons. The scutum had white enamel spots. The eyes were located on either side of the scutum, oval and slightly convex. The peritreme of female ticks was short, long and comma-shaped, with a distinct chitin thickening area (Additional File 1: Fig. S1).

The body of *Alveonasus* sp. was slightly ovoid, slightly narrowed at the foot coxa IV appendage, with anterior tip narrow, into the cone-shaped apical process, posterior margin broadly rounded. The body color was red-brown, foot color slightly light. The surface was slightly elevated anteriorly and uneven posteriorly. The epidermis was wrinkled with numerous star-shaped pits. There are a pair of long disc pits parallel and close to each other on the dorsal side of the body and the middle part of the anterior part of the ventral part, and three pairs of round disc pits on both sides of the posterior part, with a few scattered short hairs on the epidermis. (Additional File 1: Fig. S2).

### Mitochondrial genome structure and nucleotide composition

**Fig. 1:**
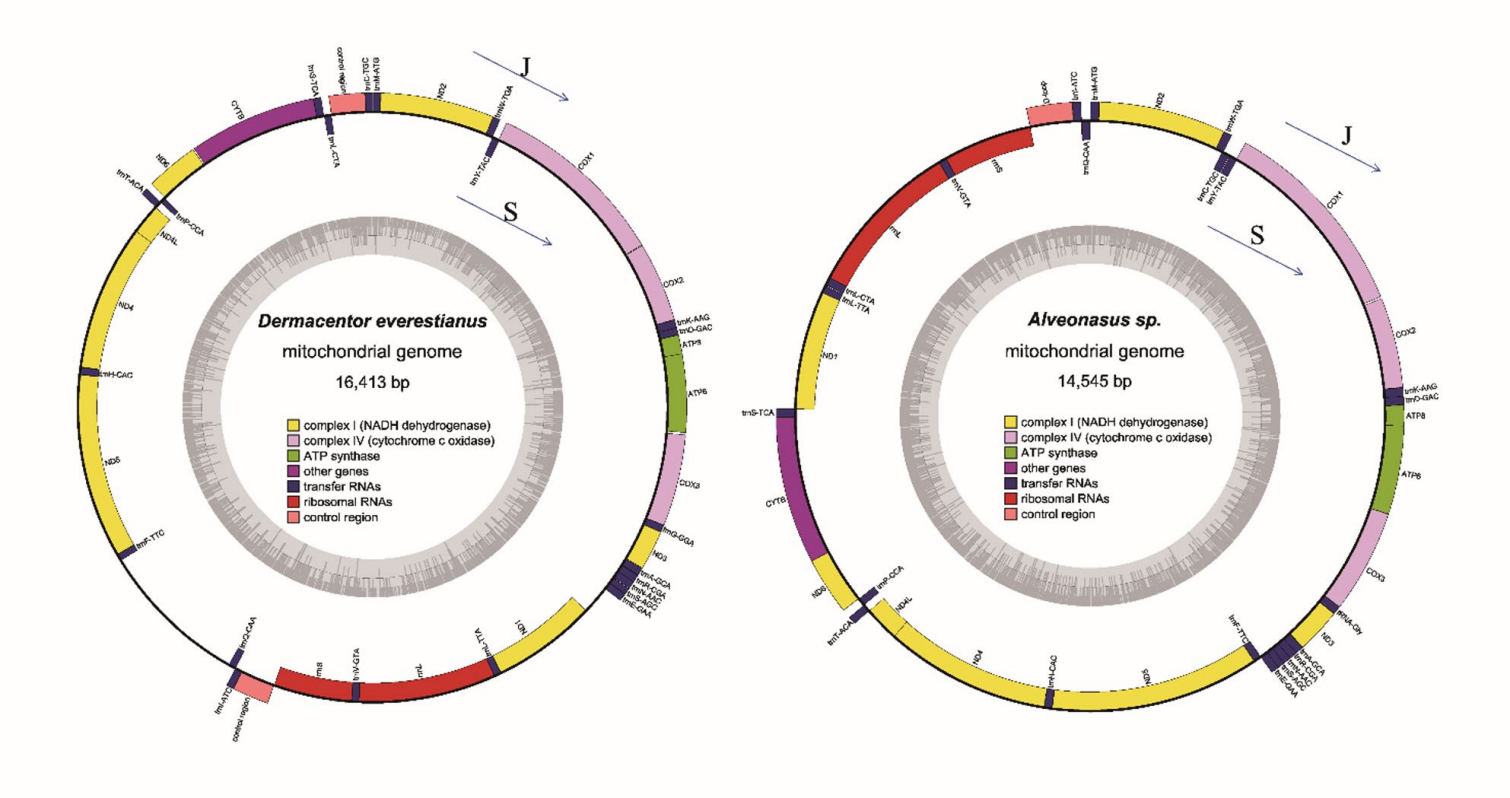
Mitochondrial genome structure of *D. everestianus* and *Alveonasus* sp. Different colors indicate different types of genes and regions

The data reported in this paper have been deposited in the NCBI GenBank (National Center for Biotechnology Information, https://www.ncbi.nlm.nih.gov). The complete mitochondrial genomes of *D. everestianus* (Accession numbers: PV023982) and *Alveonasus* sp. (Accession numbers: PV023981) are both typical circular, double-stranded structures, with total lengths of 16,413 bp and 14,545 bp, respectively (Fig. 1). *Dermacentor everestianus* is composed of 37 genes (13 PCGs, 22 tRNA genes, and 2 rRNA genes) and 2 control regions (CR). *Alveonasus* sp. comprises 37 genes (13 PCGs, 22 tRNA genes, and 2 rRNA genes) and one D-loop.

The nucleotide composition of *D. everestianus* is A = 38.38%, T = 40.3%, C = 11.67%, and G = 9.64%; the nucleotide composition of *Alveonasus* sp. is A = 38.03%, T = 33.57%, C = 20.03%, and G = 8.37%. Both species exhibit high AT content in their mitogenomes, with 78.68% for *D. everestianus* and 71.60% for *Alveonasus* sp. The AT-skew of *D. everestianus* (-0.024) is negative, while that of *Alveonasus* sp. (0.062) is positive (Table 2). *D. everestianus* exhibited 23 intergenic spacer regions and gene overlap sites, whereas *Alveonasus* sp. displayed 22 such sites. The longest intergenic spacer, 259 bp, is located between *trnE* and *ND1*, and the longest gene overlap, 24 bp, occurs between *trnN* and *trnS1* (Tables 3 and 4).

**Table 2:** Base composition of the mitochondrial genome in *D. everestianus* and *Alveonasus* sp.

Species	Region	A content	T content	C content	G content	AT content	CG content	AT-skew	CG-skew
*Dermacentor everestianus*	Complete sequence	38.38	40.3	11.67	9 0.64	78.68	21.31	-0.024	-0.095
	tRNAs	41.55	38.75	10.42	9.27	80.3	19.69	0.035	-0.058
	rRNAs	39.2	41.9	10.96	7.94	81.1	18.9	-0.033	-0.160
	Control region	37.07	37.88	12.58	12.47	74.95	25.05	-0.011	-0.004
	*ND2*	37.38	49.22	8.31	5.09	86.66	13.4	-0.137	-0.240
	*COX1*	30.8	40.16	15.4	13.65	70.96	29.05	-0.132	-0.060
	*COX2*	36.26	39.97	13.22	10.55	76.23	23.77	-0.049	-0.112
	*ATP8*	41.36	43.83	11.11	3.7	85.19	14.81	-0.029	-0.500
	*ATP6*	31.23	48.5	11.26	9.01	79.73	20.27	-0.217	-0.111
	*COX3*	28.15	46.14	12.98	12.72	74.29	25.7	-0.242	-0.010
	*ND3*	32.45	51.62	6.78	9.14	84.07	15.92	-0.228	0.148
	*ND1*	33.87	44.09	9.48	12.57	77.96	22.05	-0.131	0.140
	*ND5*	33.76	47.1	8.7	10.45	80.86	19.15	-0.165	0.091
	*ND4*	32.5	46.32	9.42	11.77	78.82	21.19	-0.175	0.111
	*ND4L*	32.97	49.28	4.71	13.04	82.25	17.75	-0.198	0.469
	*ND6*	37.11	49.33	9.56	4.00	86.44	13.56	-0.141	-0.410
	*CYTB*	32.32	44.97	12.37	10.34	77.29	22.71	-0.164	-0.089
*Alveonasus sp.*	Complete sequence	38.03	33.57	20.03	8.37	71.6	28.4	0.062	-0.411
	tRNAs	39.79	35.86	14.55	9.80	75.65	24.35	0.052	-0.195
	rRNAs	38.81	35.62	18.36	7.21	74.43	25.57	0.043	-0.436
	Control region	34.69	39.36	15.16	10.79	74.05	25.95	-0.063	-0.168
	*ND2*	32.81	42.24	18.24	6.71	75.05	24.95	-0.126	-0.462
	*COX1*	29.95	34.7	22.09	13.26	64.65	35.35	-0.073	-0.250
	*COX2*	34.81	33.33	22.07	9.78	68.14	31.85	0.022	-0.386
	*ATP8*	45.51	33.33	17.95	3.21	78.84	21.16	0.154	-0.697
	*ATP6*	31.39	39.01	22.12	7.47	70.4	29.59	-0.108	-0.495
	*COX3*	28.15	46.14	12.98	12.72	74.29	25.71	-0.242	-0.010
	*ND3*	31.27	40.71	20.94	7.08	71.98	28.02	-0.131	-0.495
	*ND5*	27.25	45.68	7.68	19.39	72.93	27.07	-0.253	0.433
	*ND4*	24.89	47.74	7.16	20.21	72.63	27.37	-0.315	0.477
	*ND4L*	24.73	50.54	3.94	20.79	75.27	24.73	-0.343	0.681
	*ND6*	34.94	40.23	20.46	4.37	75.17	24.83	-0.070	-0.648
	*CYTB*	30.23	37.65	22.99	9.14	67.88	32.13	-0.109	-0.431
	*ND1*	24.34	46.56	6.56	22.54	70.9	29.1	-0.313	0.549

**Table 3:** Locations and start/stop codons of mitochondrial genes in *D. everestianus*

Name	Start	Stop	Strand	Length(bp)	Intergenic nucleotides(bp)	Codons
*trnM*	1	65	J	65	0	
*ND2*	66	1028	J	963	-1	ATT/TAA
*trnW*	1028	1090	J	63	5	
*trnY*	1094	1157	N	64	-8	
*COX1*	1150	2688	J	1539	6	ATT/TAA
*COX2*	2693	3365	J	673	0	ATG/TAA
*trnK*	3366	3435	J	70	-2	
*trnD*	3434	3496	J	63	0	
*ATP8*	3497	3658	J	162	-7	ATA/TAA
*ATP6*	3652	4317	J	666	18	ATG/TAA
*COX3*	4334	5111	J	778	0	ATG/TAA
*trnG*	5112	5174	J	63	-3	
*ND3*	5172	5510	J	339	6	ATA/TAA
*trnA*	5515	5579	J	65	4	
*trnR*	5582	5645	J	64	-2	
*trnN*	5644	5711	J	68	6	
*trnS1*	5716	5771	J	56	2	
*trnE*	5772	5836	J	65	259	
*ND1*	6094	7033	N	939	0	ATG/TAA
*trnL2*	7033	7096	N	64	0	
*rrnL*	7097	8329	N	1233	0	
*trnV*	8330	8394	N	65	0	
*rrnS*	8395	9098	N	704	0	
*Control region*	9099	9407	J	309	0	
*trnI*	9408	9471	J	64	3	
*trnQ*	9473	9538	N	66	0	
*trnF*	10,882	10,941	N	60	3	
*ND5*	10,943	12,598	N	1656	0	ATT/TAA
*trnH*	12,599	12,664	N	66	4	
*ND4*	12,667	13,983	N	1317	-7	ATG/TAA
*ND4L*	13,977	14,252	N	276	4	ATG/TAA
*trnT*	14,255	14,315	J	61	0	
*trnP*	14,316	14,364	N	49	8	
*ND6*	14,371	14,820	J	450	13	ATA/TAA
*CYTB*	14,832	15,914	J	1083	-1	ATG/TAG
*trnS2*	15,914	15,979	J	66	4	
*trnL1*	15,982	16,044	N	63	0	
Control region	16,045	16,349	J	305	0	
*trnC*	16,350	16,410	J	61	0	

*J* Major-coding strand, *N *Minor-coding strand. In the column of “Gene interval”, the negative numbers indicated overlapping length

**Table 4:** Locations and start/stop codons of mitochondrial genes in *Alveonasus* sp.

Name	Start	Stop	Strand	Length(bp)	Intergenic nucleotides(bp)	Codons
*trnM*	1	66	J	66	0	
*ND2*	67	1020	J	954	0	ATT/TAA
*trnW*	1019	1082	J	64	-8	
*trnC*	1075	1137	N	63	7	
*trnY*	1143	1206	N	64	-8	
*COX1*	1199	2737	J	1539	14	ATT/TAA
*COX2*	2750	3425	J	676	0	ATG/TAA
*trnK*	3426	3493	J	68	-2	
*trnD*	3492	3555	J	64	0	
*ATP8*	3556	3711	J	156	-7	ATT/TAA
*ATP6*	3705	4373	J	669	-1	ATG/TAA
*COX3*	4373	5149	J	777	0	ATG/TAA
*trnG*	5150	5214	J	65	0	
*ND3*	5215	5552	J	339	3	ATT/TAG
*trnA*	5554	5615	J	62	-2	
*trnR*	5614	5673	J	60	-2	
*trnN*	5672	5733	J	62	-24	
*trnS1*	5710	5788	J	79	-5	
*trnE*	5784	5845	J	62	-2	
*trnF*	5844	5906	N	63	0	
*ND5*	5907	7572	N	1666	0	ATT/TAA
*trnH*	7573	7634	N	62	4	
*ND4*	7637	8962	N	1326	-7	ATG/TAG
*ND4L*	8956	9234	N	279	4	ATG/TAA
*trnT*	9237	9296	J	60	0	
*trnP*	9297	9360	N	64	4	
*ND6*	9363	9797	J	435	-1	ATT/TAA
*CYTB*	9797	10,901	J	1105	0	ATG/TAA
*trnS2*	10,902	10,964	J	63	-2	
*ND1*	10,963	11,907	N	945	-3	ATA/TAG
*trnL2*	11,905	11,965	N	61	7	
*trnL1*	11,971	12,036	N	66	0	
*rrnL*	12,037	13,300	N	1264	0	
*trnV*	13,301	13,362	N	62	0	
*rrnS*	13,363	14,069	N	707	0	
Control region	14,070	14,413	J	344	0	
*trnI*	14,414	14,478	J	65	-3	
*trnQ*	14,476	14,542	N	67	0	

### Protein-coding genes and codon usage

Both *D. everestianus* and *Alveonasus* sp. possessed 13 typical PCGs in their complete mitogenomes, including two ATP genes (*ATP6* and *ATP8*), four cytochrome genes (*CYTB* and *COX1-3*), and seven NADP genes (*ND4L* and *ND1-6*). The sizes of the PCG regions were 10,841 bp for *D. everestianus* and 10,926 bp for *Alveonasus* sp. The PCGs of *D. everestianus* were initiated by ATT (*ND2*, *COX1*, *ND5*), ATG (*ATP6*, *COX2*, *COX3*, *ND1*, *ND*4, *ND4L*, *CYTB*), or ATA (*ATP8*, *ND3*, *ND6*), *CYTB* uses the stop codon TAG, and all others are TAA. The PCGs of *Alveonasus* sp. started with ATT (*ND2*, *COX1*, *ATP8*, *ND3*, *ND5*, *ND6*), ATG (*COX2*, *ATP6*, *COX3*, *ND4*, *ND4L*, *CYTB*), and only *ND1* with ATA. *ND3*, *ND4*, and *ND1* used stop codons (TAG), while the majority ended with TAA (Tables 3 and 4).

**Fig. 2:**
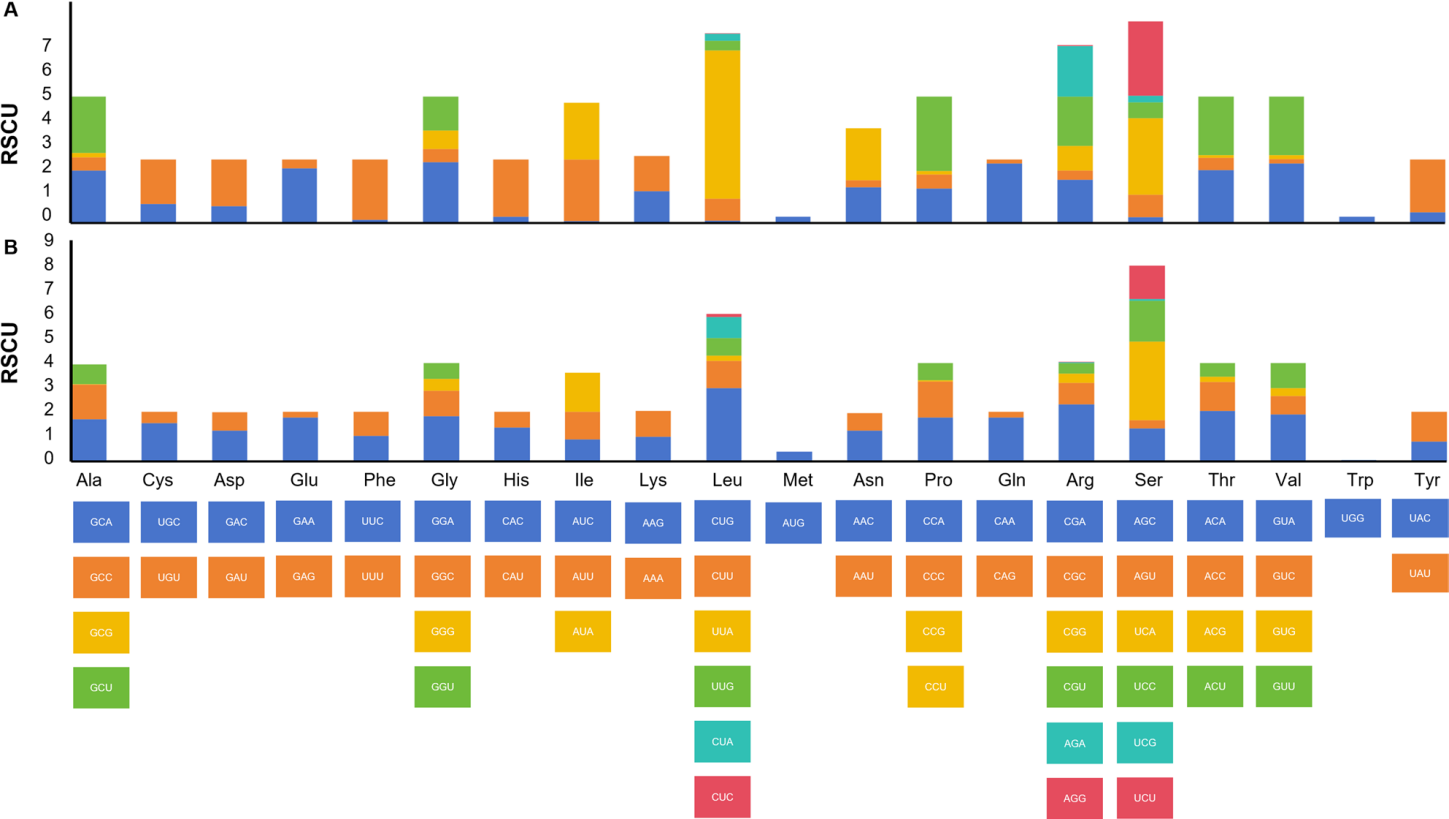
Relative synonymous codon usage (RSCU) of codons. **A** D*. everestianus* and **B** *Alveonasus* sp. The box below the bar chart represents all codons encoding each amino acid, and the height of the column above represents the sum of all RSCU values

The codon usage pattern and nucleotide composition offer a crucial theoretical foundation for understanding the genetic manipulation of mitochondrial genomes. Both *D. everestianus* and *Alveonasus* sp. exhibit specific codon usage preferences, with 61 different codons identified across the two species. *Dermacentor everestianus* utilizes 29 codons, while *Alveonasus* sp. employs 31 codons, indicating similar codon usage preferences. The most frequently used amino acids were leucine (Leu), accounting for 13.23% in *D. everestianus* and 15.66% in *Alveonasus* sp., and serine (Ser), at 9.69% and 7.05%, respectively. Tryptophan (Trp) was the least used amino acid, representing 2.26% in *D. everestianus* and 2.74% in *Alveonasus* sp. (Fig. 2).

### Nucleotide diversity (Pi) and nonsynonymous (Ka)/synonymous (Ks) mutation rate ratios

**Fig. 3:**
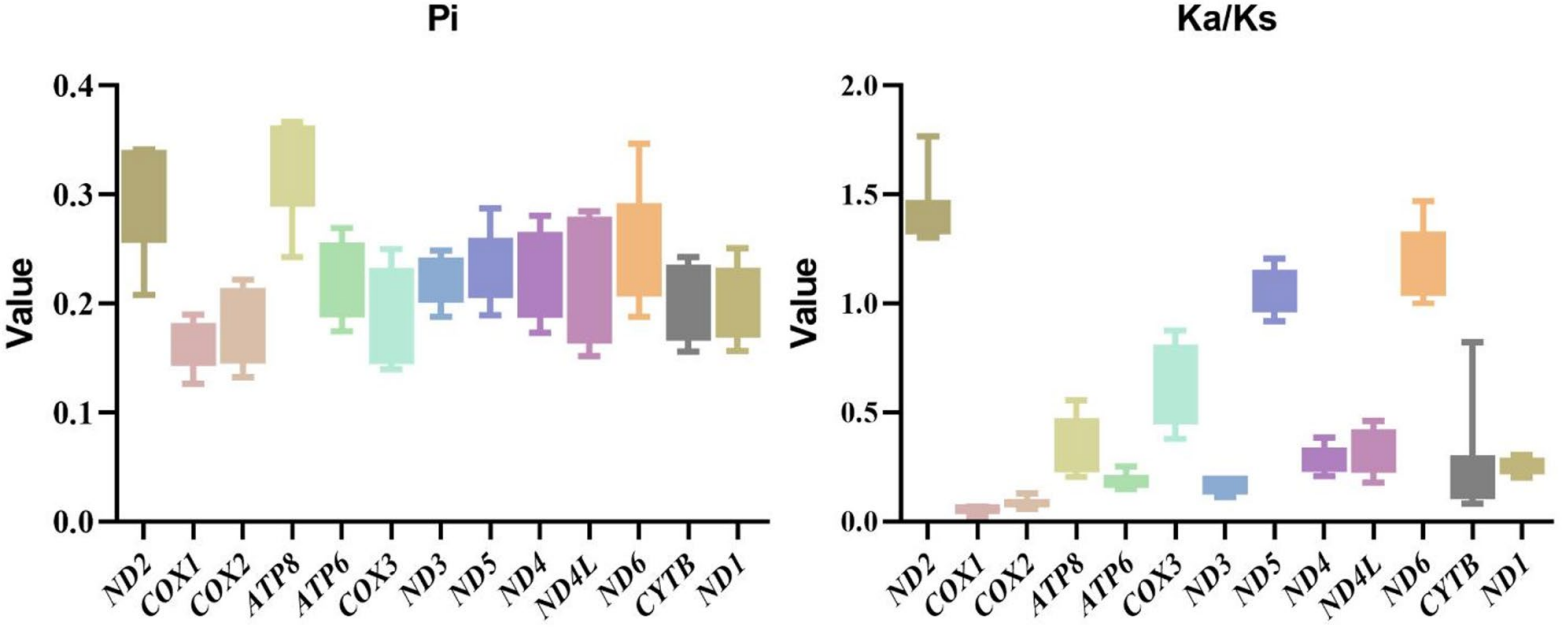
Nucleotide diversity (Pi) and nonsynonymous (Ka)/synonymous (Ks) mutation rate ratios of 13 protein-coding genes (PCGs) within mitochondrial genomes of the tick

The nucleotide diversity (Pi) of the mitochondrial genomes among the tick species was evaluated (Fig. 3), with values ranging from 0.161 (*COX1*) to 0.331 (*ATP8*). Among the PCGs, *ATP8* (0.331), *ND2* (0.293), and *ND6* (0.246) displayed the highest Pi values, indicating greater variability. Conversely, *COX1* (0.161), *COX2* (0.181), and *COX3* (0.189) exhibited the lowest Pi values, marking them as the most conserved genes. The ratios of nonsynonymous (Ka)/synonymous (Ks) mutation rates were below 1, suggesting that the PCGs are subject to purifying selection. The *COX1* gene, with the lowest evolutionary rate (Ka/Ks = 0.056), is the most conserved, while *ND2* (1.413) and *ND5* (1.045) exhibited relatively higher evolutionary rates. The Ka/Ks analysis indicated that *COX1* is the most suitable gene for DNA barcoding and initial phylogenetic analysis of ticks, making it ideal for species identification.

### Transfer and ribosomal RNA genes

Both *D. everestianus* and *Alveonasus* sp. possess 22 tRNAs. Their secondary structures are displayed in Fig. S3. The total length of the tRNAs in *D. everestianus* is 1,391 bp, with individual tRNA lengths ranging from 49 bp to 70 bp (Table 2). For *Alveonasus* sp., the total length is 1,285 bp, ranging from 60 bp to 79 bp (Table 3). Most tRNAs exhibit typical cloverleaf structures (Additional File 2: Fig. S3), except for tRNAPhe, tRNACys, and tRNASer1 in *D. everestianus*, which lack the dihydrouridine (DHU) arm. In *Alveonasus* sp., only tRNASer1 lacks the DHU arm. In addition to the typical base pairs (A-U and C-G), both species show base mismatches in the aminoacyl arms of some tRNA genes. Specifically, seven noncanonical base pairs (U-G) were identified in *trnC*, *trnD*, *trnT*, *trnH*, and *trnY*.

### Cytochrome oxidase I gene analysis

**Fig. 4:**
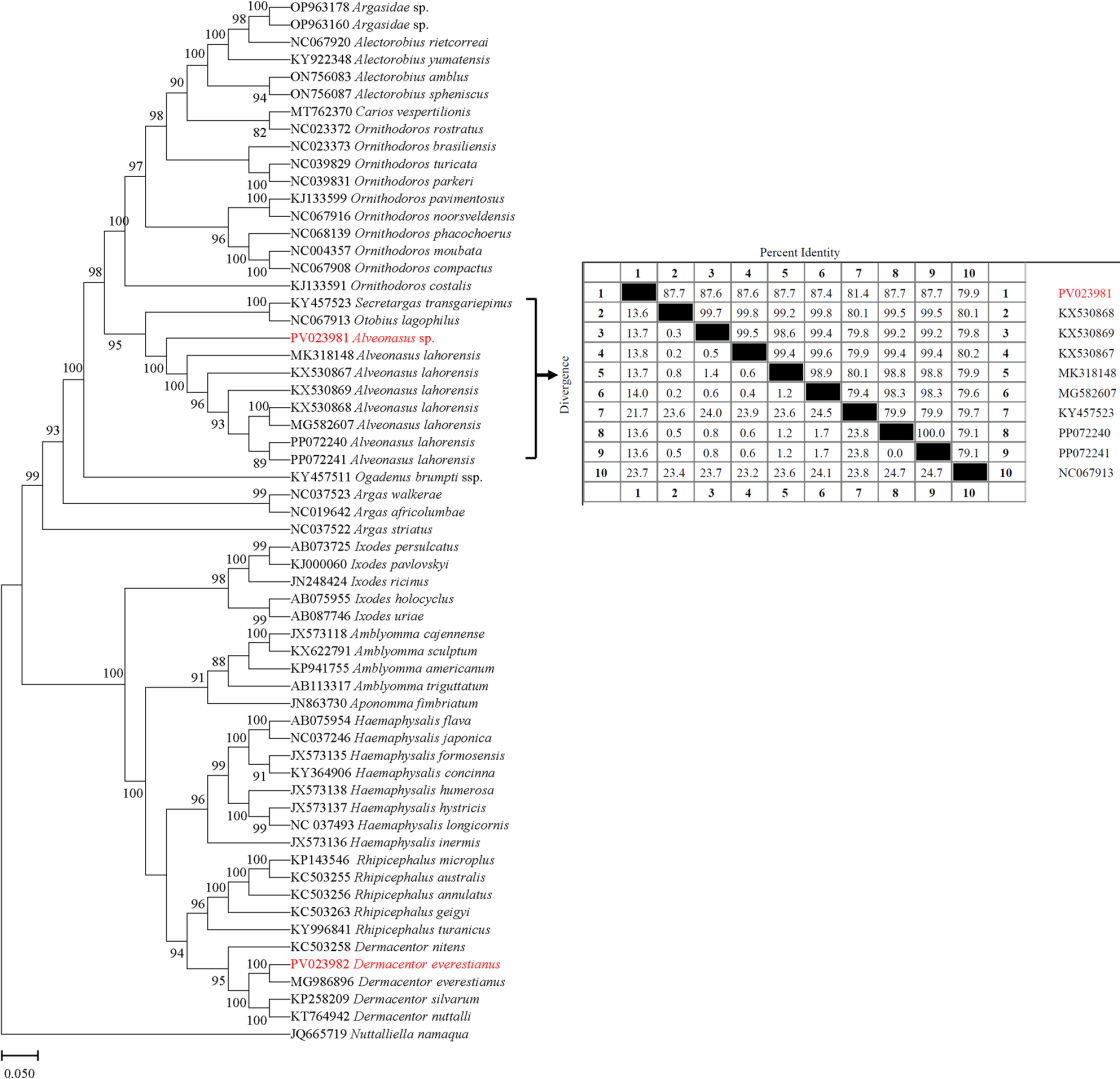
Maximum-likelihood phylogenetic tree based on cytochrome oxidase I gene. The sequence of *Nuttalliella namaqua* was used as outgroup. The bootstrap values (1000 replicates) are shown at each node. The obtained sequences for the present study are underlined and colored in red

In the concatenated phylogenetic tree constructed based on *COI* sequences (Fig. 4), *Dermacentor everestianus* was clustered together with *Dermacentor nitens*, *Dermacentor silvarum*, and *Dermacentor nuttalli*. *Alveonasus* sp. clustered with *Secretargas transgariepinus*, *Otobius lagophilus*, and *A. lahorensis*, with sequence identities ranging from 79.9% to 87.7%. Among these, it shared the highest homology of 87.7% with *A. lahorensis* from Pakistan .

### Mitochondrial genome analysis

**Fig. 5:**
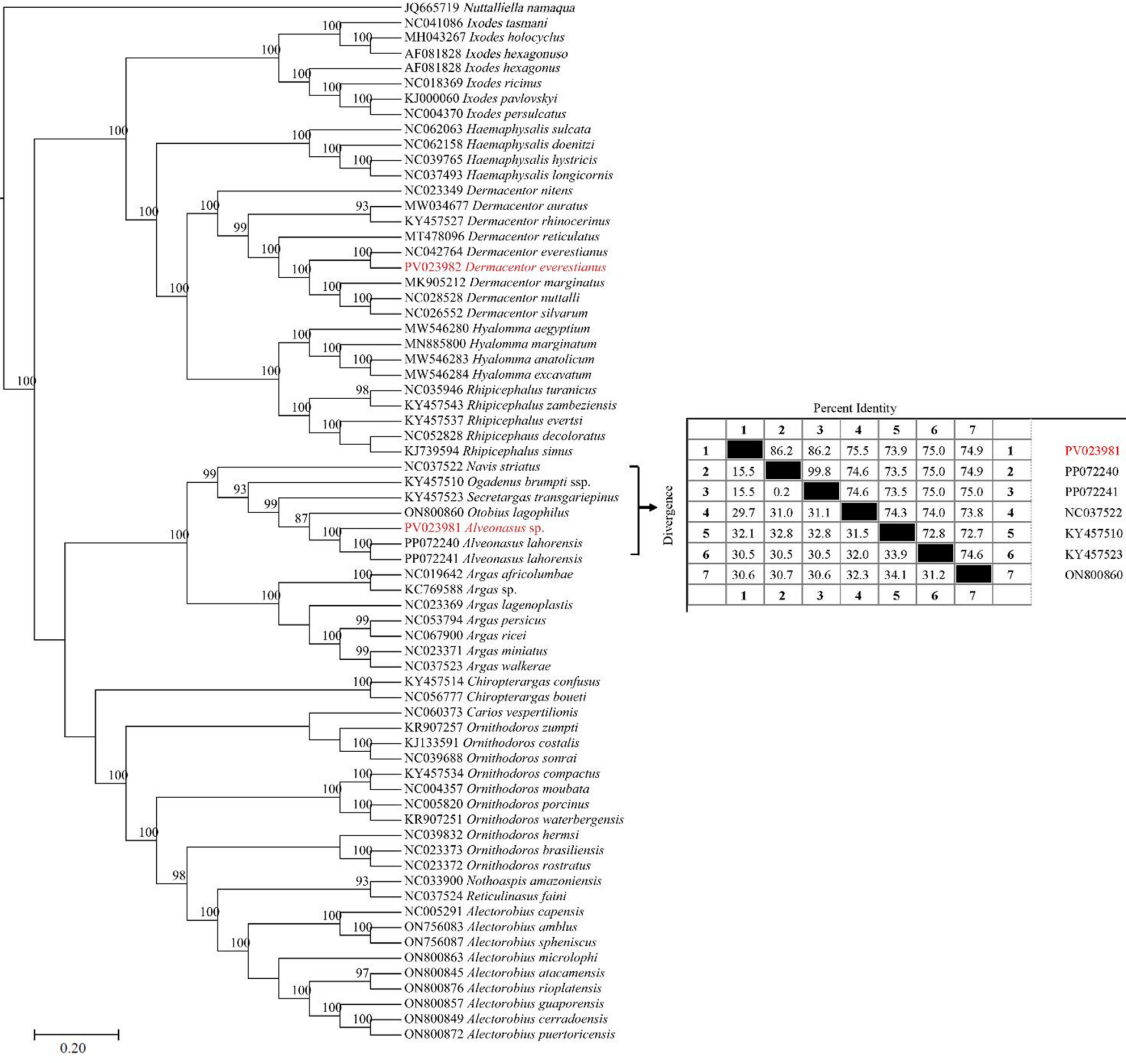
ML phylogenetic trees based on tick mitochondrial genomes of 13 concatenated protein coding nucleotide sequences. The sequence of *Nuttalliella namaqua* was used as outgroup. The bootstrap values (1000 replicates) are shown at each node. The obtained sequences are presented in red

Phylogenetic trees constructed using ML methods had topologies, with most nodes showing a support rate of 100, indicating high credibility (Fig. 5). The tree topology resolved two primary monophyletic lineages corresponding to the families Ixodidae and Argasidae, where species belonging to the same family were clustered into respective clades. The Ixodidae lineage was further subdivided into five subclades, encompassing the genera *Dermacentor*, *Haemaphysalis*, *Hyalomma*, *Ixodes*, and *Rhipicephalus*. Within the Ixodidae clade, a distinct subclade constituted a well-supported monophyletic group consisting of all *Ixodes* species. Our target species, *D. everestianus*, clustered into one branch with the clade (*Dermacentor reticulatus*+*Dermacentor everestianus*+*Dermacentor marginatus*+(*Dermacentor silvarum*+*Dermacentor nuttalli*)). In contrast, in this study, the *Alveonasus* sp. and *A. lahorensis* forms a sister-group that group in a larger clade with *Navis striatus*, *Ogadenus brumpti* ssp., *Secretargas transgariepinus* and *Otobius lagophilus*. BLASTN analysis was performed to identify all closely related sequences of *Alveonasus* sp., followed by multiple sequence alignment of all sequences within the monophyletic branch. The results revealed sequence identities ranging from 73.9% to 86.2%, with the highest identity of 86.2% observed with *A. lahorensis* from Pakistan.

## Discussion

In this study, we first sequenced and annotated the mitogenome of *D. everestianus* and *Alveonasus* sp. The mitochondrial genome of *D. everestianus* is larger than that of most *Ixodes* species, whereas the mitochondrial genome of *Alveonasus* sp. aligns with typical gene sizes found in the Argasidae family. Variations in mitochondrial genome size among ticks may be attributed to gene rearrangements and differences in the length of the control region [29]. Most metazoans have only one control region, but some species exhibit tandem repeats in this region, causing significant variation in length due to differences in repeat type and number [30]. The appearance of these tandem repeats is generally attributed to strand slippage during DNA replication [31]. In the case of *D. everestianus*, the discovery of two similar insertions between tRNA-Phe and tRNA-Gln and between tRNA-Glu and ND1 was the main factor leading to its longer genome length. The mitochondrial genome of *D. everestianus* and *Alveonasus* sp. contains the typical complement of 13 PCGs, 2 rRNA genes, and 22 tRNA genes, which is consistent with most tick mitochondrial genomes [32, 33].

At the mitochondrial genome-wide level, *D. everestianus* and *Alveonasus* sp., like most metazoan species, exhibit high A + T content, exceeding 70% [34]. In metazoan mitogenomes, A + T content generally surpasses G + C content [35–37]. Typically, A + T content is similar among species within the same family or genus. Moreover, variations in base composition are influenced by the differing habitats and survival strategies of soft and hard ticks [38]. Strand asymmetry in nucleotide composition is described using AT-skew and GC-skew values [39]. In metazoan mitogenomes, the heavy strand usually shows negative GC-skew and positive AT-skew. However, in ticks, AT-skew patterns differ between soft and hard ticks. Soft ticks typically show a positive AT-skew, while hard ticks show a positive AT-skew only in *Ixodes hexagonus* and *Ixodes uriae*, with most hard ticks showing a negative AT-skew [35]. The AT-skew and GC-skew values for *D. everestianus* and *Alveonasus* sp. in this study align with these general patterns.

The codon usage frequency in the mitochondrial genomes of *D. everestianus* and *Alveonasus* sp. is similar to that of other metazoans, with ATG as the most frequent start codon and TAA as the most common stop codon [40]. Most of the tRNAs folded into typical cloverleaf structures, except for tRNACys, tRNAPhe, and tRNASer1, which lacked the typical dihydrouridine (DHU) arm. The absence of the DHU arm in tRNASer1 appears to be an ancestral feature shared across nearly all metazoans, including ticks [41, 42]. The secondary structure of tRNACys varies among ticks. For example, *Hyalomma marginatum* [43] and *Ixodes holocyclus* were found to lack the TψC arm. Additionally, base mismatches in mitochondrial tRNAs are common in metazoans, occurring in the anticodon arm, DHU arm, amino acid acceptor arm, and TψC arm, with typical mismatches like G-U, A-C, A-G, A-A, and U-U. These mismatches generally do not impact functionality.

ML analysis of mitochondrial genes is extensively utilized in tick molecular systematics [44, 45], and the similarity between our phylogenetic results and those based on 13 PCGs suggests that using a broader set of mitochondrial genes may offer stronger support for tick classification [46]. In this study, the monophyly of subfamilies was supported by an ML tree based on 13 PCG sequences from the mitochondrial genome. The genus-level classification of hard ticks is well-established, with 18 recognized genera [47–49]. Particular focus on mitochondrial genome sequences offer the potential to elucidate disputed phylogenetic relationships within soft ticks [50, 51]. Therefore, molecular characterization based on the *COI* gene and mitochondrial genome sequencing confirmed that *Alveonasus* sp. clusters within the evolutionary branch comprising *Secretargas*, *Otobius*, and *Alveonasus*. Cladistic analysis conducted by Klompen et al. recovered *Alveonasus*, *Ogadenus*, *Proknekalia*, and *Secretargas* within a larger clade, which was designated the *Alveonasus* group [52]. Subsequently, Mans confirmed this grouping and unambiguously assigned the *Alveonasus* group to the subfamily Argasinae [53]. The family Argasidae is likely to contain multiple evolutionarily independent lineages, including members of the *Alectorobius*, *Pavlovskyella*, *Otobius*, and *Alveonasus*. Our results indicate that the *Alveonasus* sp. examined in this study represents a member of one such lineage.

## Conclusion

In this study, we sequenced the complete mitochondrial genomes of *D. everestianus* and *Alveonasus* sp., with lengths of 16,413 bp and 14,545 bp, respectively. Both *D. everestianus* and *Alveonasus* sp.contained 2 rRNA genes, 22 tRNA genes, and 13 PCGs. There are two similar insertions between tRNA-Phe and tRNA-Gln and between tRNA-Glu and ND1 in *D. everestianus*. *Dermacentor everestianus* exhibited negative AT-skews, and *Alveonasus* sp. exhibited positive AT-skews. Among all genes, *cox1* has the lowest rate of evolution. The *COI* gene, together with the complete mitochondrial genome, provided higher-resolution phylogenetic information, supporting the classification of *Alveonasus* sp. as an independent evolutionary lineage within the family Argasidae. This study not only expands the mitochondrial gene database for ticks but also offers valuable insights for the molecular classification and genetic variation analysis of ticks.

## Supplementary Information

Additional file 1: Fig S1 and Fig S2. Morphology of the adult of D. everestianus and Alveonasus sp.

Additional file 2: Fig S3.The secondary structures of the transfer RNA genes (tRNAs) in D. everestianus(A) and Alveonasus sp.(B) mitogenome.

## Data Availability

The GenBank accessions of mitogenomes generated in this study are PV023981-PV023982.
