# Supplementary material for: Complete mitochondrial and phylogenetic relationships of Dermacentor everestianus (Acari: Ixodidae) and Alveonasus sp. (Acari: Argasidae)
Source: BMC Genomics. 2026 May 21;27:678. doi: 10.1186/s12864-026-12933-2 (PMC13474809; doi:10.1186/s12864-026-12933-2)
Supplement: Supplementary file 1 [file 12864_2026_12933_MOESM1_ESM.docx]

**Fig. S1** Morphology of the adult *D. everestianus*. (A) dorsal, (B) basis capituli, (C) peritreme, (D) ventral view, (E) anus, (F) tarsus.

**Fig. S2** Morphology of the adult *Alveonasus* sp. (A) dorsal, (B) basis capituli, (C) peritreme, (D) ventral view, (E) anus, (F) tarsus.
