# Supplementary material for: Complete mitochondrial and phylogenetic relationships of Dermacentor everestianus (Acari: Ixodidae) and Alveonasus sp. (Acari: Argasidae)
Source: BMC Genomics. 2026 May 21;27:678. doi: 10.1186/s12864-026-12933-2 (PMC13474809; doi:10.1186/s12864-026-12933-2)
Supplement: Supplementary file 2 [file 12864_2026_12933_MOESM2_ESM.docx]

**Fig. S3** The secondary structures of the transfer RNA genes (tRNAs) in *D. everestianus*(A) and *Alveonasus* sp.(B) mitogenome.
